# Trends in Complementary and Alternative Medicine Use in Angina

**DOI:** 10.1093/geroni/igaf122.2896

**Published:** 2025-12-31

**Authors:** Kevin Kozakowski, Najah Khan, Audrey Shawley

**Affiliations:** Scripps Health, San Diego, California, United States; Scripps Green Hospital, San Diego, California, United States; Scripps Health, San Diego, California, United States

## Abstract

Complementary and alternative medicine (CAM) is a group of diverse medical and health care systems, practices, and products that are not generally considered part of conventional medicine. There is an increasing interest in CAM use in patients with conditions such as angina. This study strives to evaluate CAM use among adults with a history of angina. A cross-sectional analysis using data from the Integrated Public Use Microdata Series - National Health Interview Survey from 2002 was performed to assess CAM use in adults with a history of angina. This is the last year CAM data was known to be collected. Descriptive statistics and Fisher’s exact test for any single CAM modality vs demographic variables were performed. Of 1,331 respondents with a history of angina, 18 reported using at least one CAM modality. The mean age was 59-years-old with other demographic variations. The most used modalities were relaxation and herbal therapy. No other associations were identified between demographic variables and CAM use. Though the sample size was limited, this study highlights the potential trends and increased use of CAM in adults with a history of angina. Healthcare providers should be cognizant of CAM use in patients with angina and discuss the benefits and risks with their patients to ensure safe and well-coordinated care. There is a need for more rigorous research to determine the effects and long-term benefits on cardiovascular morbidity and mortality with CAM usage in patients with a history of angina.

